# Identification of a novel frameshift mutation in the *DMD* gene as the cause of muscular dystrophy in a Norfolk terrier dog

**DOI:** 10.1186/s40575-015-0019-4

**Published:** 2015-05-14

**Authors:** Christopher A Jenkins, Oliver P Forman

**Affiliations:** Kennel Club Genetics Centre, Animal Health Trust, Kentford, Newmarket, Suffolk CB8 7UU UK

**Keywords:** Dystrophin, DMD, Norfolk terrier, Muscular dystrophy, Mutation

## Abstract

**Background:**

A Norfolk terrier was referred to the Animal Health Trust neurology department with suspected dystrophin-deficient muscular dystrophy (DD-MD), which was confirmed by clinical workup and immunohistochemistry.

**Findings:**

Exon resequencing of the canine Duchenne Muscular Dystrophy (DMD) gene was undertaken to screen for potential disease causing mutations. The sequence data generated from all coding DMD exons revealed a 1 bp deletion in exon 22, causing a frameshift and premature termination of the coding sequence. Gene expression analysis indicated reduced levels of dystrophin transcript in the DD-MD case and western blot confirmed the absence of full length protein.

**Conclusions:**

The finding represents a novel mutation causing DD-MD in the dog.

**Electronic supplementary material:**

The online version of this article (doi:10.1186/s40575-015-0019-4) contains supplementary material, which is available to authorized users.

## Lay summary

In healthy skeletal muscle the dystrophin protein protects cells against damage when contracting during movement. In dystrophin deficient muscular dystrophy (DD-MD) the absence of dystrophin results in damage to the muscle. This leads to loss of mobility and reduced life expectancy.

A six month old male Norfolk terrier was referred to the Animal Health Trust neurology department, and was confirmed as having DD-MD. This was the first time the disease had been described in this breed. The aim of this study was to identify the genetic cause of the disease in this dog.

The coding exons of the dystrophin gene (*DMD*) were sequenced, in both the affected dog and its mother. A variant in exon 22 was identified which was predicted to result in the production of a truncated dystrophin protein. The lack of functional dystrophin in the affected dog was confirmed through expression analysis.

In conclusion, this study identified a novel variant in *DMD*, which is likely to be the cause of DD-MD in a Norfolk terrier dog.

## Findings

Dystrophin-deficient muscular dystrophy (DD-MD) is an X-linked disease in humans (Duchenne Muscular Dystrophy, DMD) and dogs [1]. In DD-MD the lack of functional dystrophin protein causes advancing muscle weakness, respiratory problems, and cardiomyopathy [2]. DD-MD is currently incurable, and the dog is an ideal model for the disease because the canine phenotype is very similar to that seen in humans [1]. In muscle dystrophin protects cells from shearing stress caused by contraction [3]. The absence of dystrophin damages the integrity of myofibers. Damaged muscle cells degenerate and undergo necrosis, and are ultimately replaced by adipose and fibrous tissues. This results in the loss of mobility and reduced life expectancy.

A number of pathogenic mutations have been discovered in the dystrophin gene (*DMD*) of dogs. Examples include a 4 base pair (bp) deletion in exon 65 in a Cocker spaniel [4], an intron 13 long interspersed nuclear element (LINE-1) insertion in the Pembroke Welsh Corgi [3], and a splice site mutation in the golden retriever resulting in the skipping of exon 7 [5].

**Figure 1:**
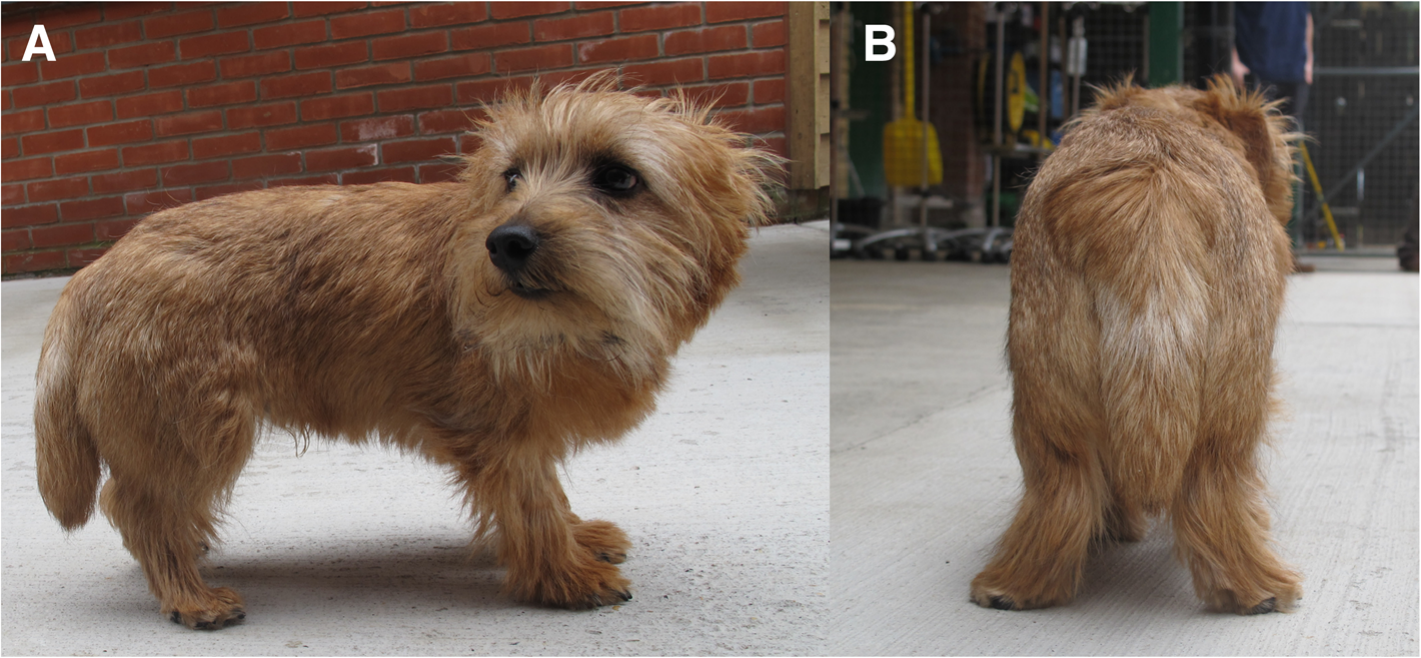
Photographs **(A and B)** depicting gross phenotype of the affected six month old male Norfolk Terrier. The affected has generalised skeletal muscle atrophy with adduction of the hocks.

A six month old Norfolk terrier was referred to the Animal Health Trust neurology department displaying clinical signs of muscular dystrophy (Figure 1), which was confirmed through a full neurological and histopathological work-up as described previously [6]. The aim of this study was to identify the causal mutation for the Norfolk terrier lacking functional dystrophin protein.

**Figure 2:**
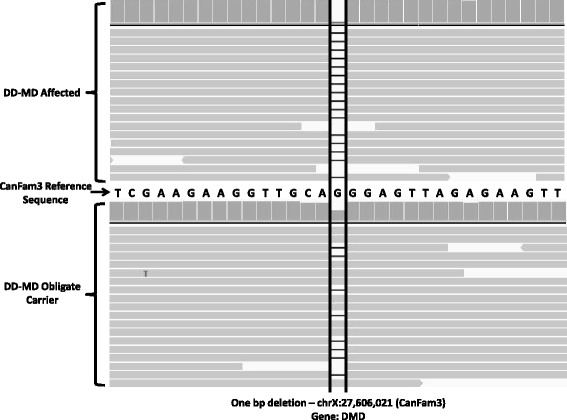
Graphical display of the 1 bp deletion in *DMD.* Exon 22 sequence read alignments for the case (top) and obligate carrier (bottom). The vertical solid grey bars represent the read depth and the horizontal grey bars represent aligned sequence reads with differences to the reference sequence highlighted. The position of the 1 bp deletion is highlighted by the two vertical black lines. The black horizontal lines in the sequence reads represent the deleted base, which is absent in case sequence reads and present in approximately half of the reads for the carrier.

Due to the large size of the dystrophin gene we focused our investigation on the 79 coding exons of *DMD*. PCR primers were designed, and amplification carried out individually for each exon using DNA from the case and its dam. The PCR products were pooled and libraries prepared for sequencing on the Ilumina MiSeq platform. The dataset of 100 bp single-end reads generated were aligned to the canine reference sequence (canfam3). Sequence read alignments were visualised in IGV [7]. A 1 bp deletion was identified in exon 22 (Figure 2). The deletion was heterozygous in the carrier (dam) and homozygous in the case. Analysis of the predicted amino acid sequence of the protein suggested that this mutation would cause a frameshift and a premature stop codon (p.Gly1029AspfsX30) [GenBank:NM001003343], resulting in protein truncation.

**Figure 3:**
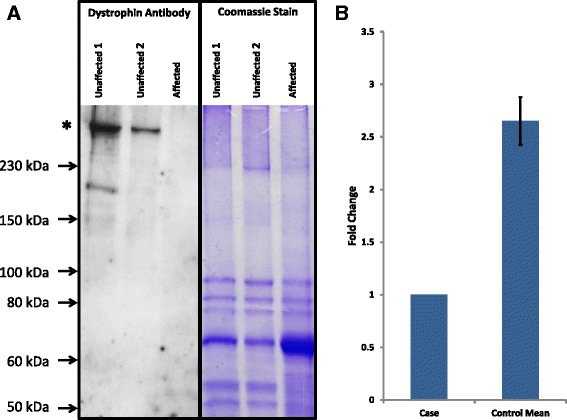
Expression Analysis. **A)** Western blot analysis of skeletal muscle samples. Left panel shows detection of dystrophin by an antibody targeting the N-terminal region (ab131315). Bands are visible for full length dystrophin (* predicted weight 427 kDa) for both of the control samples, but not the DD-MD case. The right panel shows a Coomassie blue stained SDS-PAGE protein gel which was run in parallel, showing similar distribution and intensity of bands for all three samples. **B)** Relative levels of *DMD* transcript in skeletal muscle samples demonstrated by qRT-PCR. The DD-MD case shows a 2.64 fold lower expression of *DMD* transcript in comparison to five independent skeletal muscle controls. Error bars are based on standard deviation.

To investigate the effect of the mutation on the level of *DMD* gene expression, relative quantification by quantitative reverse transcription PCR (qRT-PCR) was performed. Assays were designed for the *DMD* gene and for the ubiquitously expressed TATA box binding protein (*TBP*) gene. RNA was extracted from skeletal muscle samples obtained from one case and five control dogs. Muscle samples were not matched by breed or the muscle from which they were biopsied. qRT-PCR was carried out in triplicate, and reaction efficiencies calculated by generating a seven point, doubling dilution standard curve for both assays [8]. Results indicated a 2.64 fold reduction in muscle *DMD* expression in the DD-MD case (Figure 3b, Additional file 1), suggestive of nonsense mediated decay.

To further investigate the effect of the mutation, western blot analysis was carried out. Protein was extracted from skeletal muscle from the case, and compared to protein extracted from two unaffected dogs’ skeletal muscle. The primary antibody used for the western blot (ab131315) targeted a region close to the N-terminus of the dystrophin protein. The immunoblot showed bands for full length dystrophin in both of the controls, but not in the case (Figure 3a). This confirms that the case lacks functional full length dystrophin. No band was observed for the predicted truncated protein (approximately 122.4 kDa) caused by the mutation.

In the clinical investigation by Beltran and colleagues, immunohistochemical analysis using antibodies targeting the rod-domain and the c-terminus showed an absence of dystrophin in the skeletal muscle of the Norfolk terrier DD-MD case [6]. These findings are consistent with the frameshift mutation identified in this study, as the predicted truncated protein would lack both the rod-domain and c-terminus. The western blot antibody used in this study targeted a region close to the N-terminus (amino acids 410–450) upstream of the frameshift mutation discovered by exon resequencing. No full-length dystrophin protein was identified by immunoblot in the affected dog, consistent with the genetic findings. However, there was also no truncated dystrophin protein observed. Although the qPCR analysis showed a 2.64 fold reduction in transcript levels, gene expression was not knocked out completely. Consequently, nonsense mediated decay of the mRNA does not completely explain the absence of a shortened protein. We speculate that this may be due to cellular mechanisms controlling translation or the degradation of aberrant cellular proteins.

In this study we have successfully identified a novel frameshift mutation causing a case of DD-MD in a Norfolk terrier dog.

## Ethics

Muscle tissue from the case was taken as part of a veterinary diagnostic procedure, and a small subsection of this tissue used for the RNA and protein analyses. Muscle samples from controls were obtained post-mortem. Samples of DNA were collected by buccal swabbing which is a non-invasive procedure and does not require a U.K. home office licence. All samples were taken with full owner consent.

## Availability of supporting data

The data set supporting the results of this article is included within the article (and Additional file 1).

## Additional file

Additional file 1:
**Raw data from qRT-PCR experiments.**
