# Supplementary material for: Identification of a novel frameshift mutation in the DMD gene as the cause of muscular dystrophy in a Norfolk terrier dog
Source: Canine Genet Epidemiol. 2015 May 14;2:7. doi: 10.1186/s40575-015-0019-4 (PMC4579383; doi:10.1186/s40575-015-0019-4)
Supplement: Additional file 1: — Raw data from qRT-PCR experiments. [file 40575_2015_19_MOESM1_ESM.pdf]

Supporting Information

ID	Assay	Ct Mean	Std. Dev.	ΔCt	ΔCt Mean	ΔΔCt	Fold Change
**Case**	DMD	28.08	0.01				
**Control 1**	DMD	24.31	0.07				
**Control 2**	DMD	24.18	0.12				
**Control 3**	DMD	24.21	0.04				
**Control 4**	DMD	25.06	0.11				
**Control 5**	DMD	24.03	0.08				
**Case**	TBP	29.91	0.26	1.82	1.82	1.40	**2.64**
**Control 1**	TBP	27.64	0.20	3.33	3.23		
**Control 2**	TBP	27.41	0.03	3.23			
**Control 3**	TBP	27.34	0.05	3.13			
**Control 4**	TBP	28.13	0.08	3.07			
**Control 5**	TBP	27.39	0.11	3.36			
